# Eating behaviors, eating styles and body mass index during COVID-19 confinement in a college sample: a predictive model

**DOI:** 10.1186/s40337-022-00624-8

**Published:** 2022-07-12

**Authors:** Tamara Escrivá-Martínez, Marta Miragall, Rocío Herrero, Marta Rodríguez-Arias, Rosa M. Baños

**Affiliations:** 1 Department of Personality, Evaluation and Psychological Treatments, University of Valencia, Valencia, Spain; 2 Instituto Polibienestar, University of Valencia, Valencia, Spain; 3 CIBER de Fisiopatología de la Obesidad y la Nutrición (CIBEROBN), Instituto de Salud Carlos III, Madrid, Spain; 4 Department of Psychology and Sociology, University of Zaragoza, Zaragoza, Spain; 5 Department of Psychobiology, University of Valencia, Valencia, Spain

**Keywords:** COVID-19 confinement, Maladaptive eating styles, Binge eating, Fat intake, Body mass index, Dysfunctional eating behaviors

## Abstract

**Background:**

COVID-19 confinement affected lifestyles. There is inconclusive evidence about changes in eating patterns, and there are few studies on the impact on body mass index (BMI), the occurrence of dysfunctional behaviors (binge eating, fat intake), and the predictive role of maladaptive eating styles (emotional, external, and restrained eating).

**Objectives:**

(1) To analyze the differences in binge eating, fat intake, BMI, and maladaptive eating styles before and during COVID-19 confinement, and (2) to analyze whether maladaptive eating styles (before confinement) predicted binge eating, fat intake, and BMI during confinement.

**Methods:**

The sample consisted of 146 Spanish college students, divided into 104 females (71.2%; age: *M* = 22.20, *SD* = 2.97) and 42 males (28.8%; age: *M* = 24.74; *SD* = 3.53). All completed several dietary measures and BMI twice: before COVID-19 confinement (T1, November 2019) and during COVID-19 confinement (T2, April 2020).

**Results:**

BMI and maladaptive eating styles did not change in T2 (vs. T1). However, binge eating and fat intake decreased in T2. Emotional eating at T1 positively predicted BMI and binge eating at T2. External eating at T1 positively (and marginally) predicted fat intake at T2. Restrained eating at T1 positively predicted binge eating at T2, and negatively (and marginally) predicted BMI and fat intake at T2. The model explained 80.5% of the variance in BMI, 41.5% of the variance in binge eating, and 25.8% of the variance in fat intake during COVID-19 confinement.

**Conclusions:**

The COVID-19 confinement had a positive impact on some eating behaviors. Future policies should focus part of their prevention on maladaptive eating styles to curb dysfunctional eating behaviors and BMI problems in times of stress.

## Plain English summary

The COVID-19 confinement affected the lifestyles of the young population, especially eating behaviors. It is not yet known whether eating problems, such as binge eating and high fat intake, changed during this period in the young people. It is also not known whether the young population perceived changes in their BMI during COVID-19 confinement. In addition, eating styles (emotional eating, restrictive eating, and external eating) may be predictors of this change. The present study found that binge eating and fat intake decreased in COVID-19 confinement, and BMI and eating styles remained stable. Eating styles predicted change in these eating problems (binge eating and fat intake) and change in BMI during this period of elevated stress. Therefore, eating styles may help to understand dietary changes during times of high stress.

## Background

The lockdown due to COVID-19 helped in reducing infection rates, but it also involved a change in the population's living habits, especially affecting lifestyles [1]. Research that analyzed how the COVID-19 confinement and the pandemic situation influenced eating behaviors in general population have produced mixed results.

Several studies have shown that social isolation increases negative emotions, and food intake could be a coping strategy to deal with these unpleasant emotions [2]. Specifically, it has been observed that feelings of loneliness and boredom can lead individuals to increase food consumption [3], leading in turn to weigh gain if they do not engage in any physical activity [4]. Being at home and having large amounts of food available 24 h a day during this period may also have increased food intake. Along these lines, several studies highlighted an increase in the consumption of unhealthy foods (rich in fats and sugars) and an increase in the Body Mass Index (BMI) during confinement [5–8], although the increase in BMI occurred especially in overweight or obese individuals. In this regard, binge eating behavior -understood as the consumption of a large amount of food in a short time and loss of control during intake- [9] also increased during confinement [10]. This increase in binge eating could be explained by the stress caused during the pandemic (due to economic and social difficulties) [11]. Another line of studies pointed out that there was an increase in healthy food consumption and a decrease in fast food intake during the confinement, as individuals had more time to cook and take care of eating and physical exercise behaviors, especially in young people [6, 12]. In fact, general population reported no changes in their BMI [6, 13].

In addition to understanding whether eating behaviors changed, it seems highly important to knowing the risk factors that may predict binge eating, unhealthy food consumption (e.g., fat intake), and BMI during confinement. Knowing the risk factors may help us improve their prevention and treatment in future stressful situations. In this regard, maladaptive eating styles stand out as important risk factors: emotional eating (eating in response to negative emotions), external eating (eating in response to external cues, such as smelling delicious food), and restrained eating (eating restrictively to lose or control weight) [14]. There is strong evidence suggesting associations between maladaptive eating styles, binge eating and unhealthy food consumption [15–18].

Individuals with higher emotional eating show greater dysregulation of their emotions and may use food to cope with those negative emotions [19]. In fact, emotional eating may also directly affect BMI [20]. Studies conducted during COVID-19 confinement indicate that the way of responding emotionally to COVID-19 may have also increased the risk of dysfunctional eating behaviors. For instance, emotional eaters may have increased food consumption with the goal of seeking emotional reward in food during confinement, overriding any hunger and satiety signals from the body [21].

In addition, being an external eater could also have an influence on food consumption during COVID-19 confinement, as perceived stress may influence external eating [22]. Besides, a positive relationship has been observed between external eating and consuming higher amounts of energy and unhealthy food during regular periods [23]. In this regard, considering that large amounts of food were stored at home during COVID-19 confinement [24], and that external eaters tend to eat more in response to food properties, including contextual ones [14], it could be expected that external eaters would eat more -specially, unhealthy- food during COVID-19 confinement. In turn, restrained eaters could also have overeaten in the face of the confinement situation, as stress may increase the risk of binge eating in individuals who exhibit restrained eating behaviors [25].

To date, the effect of COVID-19 confinement on eating behaviors remain unclear as conclusions are heterogeneous depending on the study sample (e.g., overweight may be risk factor) [7, 8]. However, it should be noted that most studies only measured eating behaviors and BMI at one point in time (during COVID-19 confinement) and asked about eating behavior retrospectively [5, 6, 8, 13], which could lead to biases. To our best knowledge, there are no studies assessing changes in the eating behaviors and BMI during COVID-19 confinement and comparing to data obtained before COVID-19 confinement. Therefore, the main objective of this study was to analyze the differences in binge eating, fat intake, BMI, and maladaptive eating styles before and during COVID-19 confinement in a sample of college students. Furthermore, considering the literature mentioned, it can be assumed that maladaptive eating styles could influence binge eating behavior, fat intake, and BMI during COVID-19 confinement. However, the predictive role of maladaptive traits of eating styles in these variables has not been studied. Therefore, the secondary objective of this study was to analyze whether maladaptive eating styles (emotional, external, and restrained eating) assessed before COVID-19 confinement predicted binge eating, fat intake, and BMI during COVID-19 confinement. We hypothesized changes in binge eating, fat intake and BMI during COVID-19 confinement, and we expected that these variables would be affected by the maladaptive eating styles. However, we did not specified hypothesis in any direction due to the exploratory nature of this study.

## Methods

### Participants

The sample of the present study consisted of 146 college Spanish students, divided in 104 women (71,2%; age: *M* = 22.20; *SD* = 2.97) and 42 males (28,8%; age: *M* = 24.74; *SD* = 3.53). Before COVID-19 confinement, the mean BMI^1^ was 22.66 (SD = 2.92), and 6.9% were underweight, 75.17% were normal, 16.55% were overweight, and 1.38% were obese. During COVID-19 confinement, the mean BMI was 22.92 (*SD* = 3.35), and 6.9% were underweight, 71.72% were normal, 18.62% were overweight, and 2.76% were obese.

Inclusion criteria were: age between 18 and 30 years old, living in Spain during COVID-19 confinement (April, 2021), and being enrolled in the study conducted in November, 2019 [27] . Exclusion criteria were: having an eating disorder, having a medical condition that may affect eating behavior or mood, and having a diagnosis of severe mental disorder.

### Measures

*Socio-demographic and anthropometric characteristics. *Participants included information about their sex, age, marital status, weight, and height.

*Eating styles: Dutch Eating Behavior Questionnaire (DEBQ* [14, 28]*)*. This questionnaire assessed three different eating styles: emotional eating, external eating, and restrained eating. Higher scores mean greater scores in emotional, external, and restrained eating behavior. In this study, the internal consistency was adequate (α = 0.953 for emotional eating, α = 0.885 for external eating, and α = 0.920 for restrained eating).

*Binge eating: Binge Eating Scale (BES* [29, 30]*).* This questionnaire identifies the symptoms associated with binge eating (eating large amounts of food in a short time and the feel of losing control). Higher scores indicate greater binge eating. In this study, the internal consistency was adequate (α = 0.868 for before COVID-19 confinement, and α = 0.854 during COVID-19 confinement).

*Fat intake: Short Fat Questionnaire (SFQ* [31]*).* This scale evaluates the weekly frequency of intake of high-fat food. Higher scores indicate a higher frequency of fat food intake. In this study, the internal consistency was adequate (α = 0.794 before COVID-19 confinement, and α = 0.826 during COVID-19 confinement).

### Design and procedure

Participants belonging to a previous study [27] were contacted via email and were invited to participate. Those who agreed to participate in the current study signed an informed consent, in which also agreed to retrieve their previous information. The first assessment was completed in November 2019 (T1; non-pandemic period in Spain), and the second assessment was completed in April 2020 (T2; strict COVID-19 confinement period in Spain). All the study was conducted using the Lime Survey platform of the University of Valencia.

### Ethical considerations

The study was performed under the ethical standards of the Declaration of Helsinki [32] and approved by the ethical committee of the University of Valencia (Number: 1821046).

### Data analyses

To decide which statistical test to compute to satisfy the first aim of the study, normality assumptions were tested on the difference of pairs in each of the variables of interest using Shapiro–Wilk tests. If the assumption was met, a paired t-test was conducted. Otherwise, a robust paired t-test was carried out, concretely Yuen's test on trimmed means for dependent samples with a 0.2 level of trim [33]. Effect sizes were computed in the form of Cohen's *d* (*d*; [34]) when assumptions were met, and in the form of robust Cohen's *d* (*d*_*R*_; [35]) in case they were not.

**Fig. 1:**
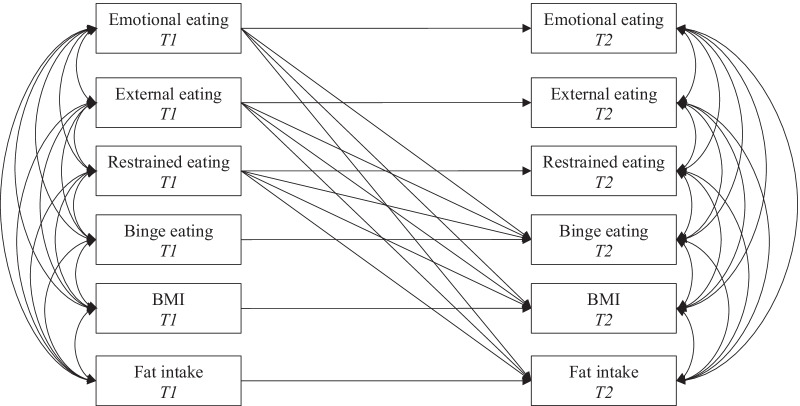
Theoretical proposed model

Regarding the second aim of the study, a Structural Equation Model (SEM) was tested to examine whether maladaptive eating styles before COVID-19 confinement (T1) had an effect on binge eating, fat intake, and BMI during COVID-19 confinement (T2). A crossed-lagged autoregressive panel model was proposed. The theoretical model is shown in Fig. 1. The Maximum Likelihood Robust (MLR) method of estimation was used. Model fit was examined using chi-square statistic (χ^2^), Comparative Fit Index (CFI), Weighted Least Squares Mean and Variance corrected (WLSMV), and Standardized Root Mean Square Residual (SRMR) [36].

Bivariate analyses were performed using R [37]. Additional to the system library, R packages employed in the analyses were: WRS2 [38] and LRS [39]. Multivariate analyses were performed using MPlus 8.6 [40].

## Results

### Differences in BMI, binge eating, fat intake, and eating styles, before and during COVID-19 confinement

The Shapiro–Wilk test showed the score difference between T1 and T2 did not follow a normal distribution for BMI, binge eating and fat intake; however, the distribution for all three eating styles were normal (see Table 1).

**Table 1:** Results of the Shapiro–Wilk normality tests

Variable	*W*	*p*
Emotional eating	.989	.347
External eating	.987	.217
Restrained eating	.985	.125
Body mass index	.922	< .001
Binge eating	.976	.013
Fat intake	.917	< .001

Due to the non-normality for the difference of pairs in binge eating, fat intake and BMI, Yuen's test for dependent samples was performed in these cases. Regarding BMI, no differences were found between T1 (*M* = 22.66, *SD* = 2.92) and T2 (*M* = 22.92, *SD* = 3.35), *t*(87) = -0.68, *p* = 0.499, *d*_*R*_ = 0.21. However, regarding binge eating and fat intake, individuals exhibited a reduction in their binge eating habits during COVID-19 confinement compared to the time before the confinement (T1: *M* = 7.67, *SD* = 6.33; T2: *M* = 6.53, *SD* = 5.82; *t*(87) = 2.07, *p* = 0.041, *d*_*R*_ = 0.23), and also decreased their fat intake during confinement (T1: *M* = 20.39, *SD* = 8.17; T2: *M* = 22.21, *SD* = 7.70; *t*(87) = 2.75, *p* = 0.007, *d*_*R*_ = 0.36).

Regarding maladaptive eating styles, t-tests were computed to examine differences in emotional, external, and restrained eating before and during the COVID-19 confinement. Results showed no statistically significant differences for emotional eating before an during the restrictions (T1: *M* = 25.27, *SD* = 9.90; T2: *M* = 25.42, *SD* = 10.44; *t*(143) = -0.42, *p* = 0.672, *d* = 0.035). For external eating, no statistically significant differences emerged (T1: *M* = 29.40, *SD* = 7.20; T2: *M* = 28.77, *SD* = 7.17; *t*(143) = 1.20, *p* = 0.231, *d* = 0.10). This was also the case for restrained eating (T1: *M* = 21.31, *SD* = 7.88; T2: *M* = 21.71, *SD* = 7.93; *t*(143) = -0.83, *p* = 0.408, *d* = 0.069).

### The predicting role of maladaptive eating styles (before COVID-19 confinement) in BMI, binge eating, fat intake, and eating styles (during COVID-19 confinement)

The theoretical model shown in Fig. 1 was established and tested. Model results indicated an excellent fit of model to the data: χ^2^(21) = 22.04, *p* = 0.397, CFI = 0.99, RMSEA = 0.018 (90% CI 0.000–0.073), SRMR = 0.036. Standardized model effects are shown in Table 2. Correlations among exogenous variables can be shown in Table 3 and correlations among dependent variables are displayed in Table 4.

**Table 2:** Coefficients, standard errors, and p-values of the tested model

	Standardized coefficient	Standard error	p-value
*Emotional eating T2*			
Emotional eating T1	.692	.046	< .001
*External eating T2*			
External eating T1	.639	.052	< .001
*Restrained eating T2*			
Restrained eating T1	.709	.044	< .001
*BMI T2*			
BMI T1	.897	.024	< .001
Emotional eating T1	.105	.050	.036
External eating T1	− .014	.048	.766
Restrained eating T1	− .080	.044	.071
*Binge eating T2*			
Binge eating T1	.418	.082	< .001
Emotional eating T1	.159	.078	.041
External eating T1	.046	.070	.512
Restrained eating T1	.165	.057	.004
*Fat intake T2*			
Fat intake T1	.425	.095	< .001
Emotional eating T1	− .094	.059	.107
External eating T1	.138	.082	.090
Restrained eating T1	− .102	.053	.055

**Table 3:** Correlation coefficients and standard errors among exogenous variables

		(1)	(2)	(3)	(4)	(5)
(1) Emotional eating T1		1				
(2) External eating T1	Coefficient	.485**	1			
	Standard error	.067				
(3) Restrained eating T1	Coefficient	.315**	.153	1		
	Standard error	.082	.084			
(4) BMI T1	Coefficient	.052	− .008	.152*	1	
	Standard error	.093	.094	.073		
(5) Binge eating T1	Coefficient	.587**	.433**	.472**	.245*	1
	Standard error	.059	.075	.062	.087	
(6) Fat intake T1	Coefficient	.126	.370**	− .190*	− .013	− .007
	Standard error	.095	.074	.078	.089	.092

*p < .05; **p < .001

**Table 4:** Correlation coefficients and standard errors among dependent variables

		(1)	(2)	(3)	(4)	(5)
(7) Emotional eating T2		1				
(8) External eating T2	Coefficient	.529**	1			
	Standard error	.067				
(9) Restrained eating T2	Coefficient	.336**	.271*	1		
	Standard error	.081	.085			
(10) BMI T2	Coefficient	− .008	− .031	.109	1	
	Standard error	.071	.075	.073		
(11) Binge eating T2	Coefficient	.553**	.513**	.344**	.015	1
	Standard error	.062	.066	.079	.080	
(12) Fat intake T2	Coefficient	.134	.505**	− .104	.002	.224*
	Standard error	.092	.082	.068	.065	.080

*p < .05; **p < .001

Regarding the effects of eating styles before COVID-19 confinement (T1) on binge eating, fat intake, and BMI during COVID-19 confinement (T2), results showed that emotional eating in T1 had statistically significant effects on both BMI (β = 0.105, *p* = 0.036) and binge eating (β = 0.159, *p* = 0.041) at T2. Restrained eating in T1 also displayed a statistically significant effect on binge eating (β = 0.165, *p* = 0.004) in T2 and a marginally significant effect on BMI (β = − 0.080, *p* = 0.071) in T2. Lastly, both restrained eating (β =0.102, *p* = 0.055) and external eating (β = 0.138, *p* = 0.090) before COVID-19 confinement had marginally significant effects on fat intake at T2.

The model explained an 80.5% of variance in BMI, a 41.5% of binge eating, and a 25.8% of fat intake during COVID-19 confinement. Moreover, eating styles scores before COVID-19 confinement explained the variance of eating styles during COVID-19 confinement to different extents: 47.9% for emotional eating, 40.8% for external eating, and 50.3% for restrained eating.

## Discussion

The current longitudinal study compares binge eating, fat intake, BMI, and maladaptive eating behaviors before and during the strict COVID-19 confinement in a sample of Spanish college students. Furthermore, the predictive role of maladaptive eating styles (emotional, external, and restrained eating) before the COVID-19 confinement was also analyzed.

The first objective was focused on analyzing the differences in binge eating, fat intake, BMI, and maladaptive eating behaviors between before and during COVID-19 confinement. Regarding BMI, no significant changes were observed. This stability in BMI could be related to the fact that our sample adopted healthy lifestyle habits during COVID-19 confinement. Individuals decreased their binge eating and fat intake during the lockdown. Although this finding could be surprising -since it could be reasonable to think that people eat worse in very stressful times- [24], this is in line with other studies that showed that people adopted healthy habits during this COVID-19 confinement [41]. One possible explanation is that the consumed food was healthier since it was homemade, and there was less access to restaurants and ultra-processed foods [6, 42]. In addition, some evidence shows that younger people adhered better to healthy eating patterns than older people [6]. Furthermore, most of the participants had a normal weight condition (75%) before COVID-19 confinement, and maybe, we can speculate that these dysfunctional eating behaviors only occurred in overweight or obese young people. Along this lines, Di Renzo et al. [6] pointed out that people with normal weight have greater adherence to healthy eating guidelines than overweight people.

As regards the changes in emotional, external, and restrained eating, no changes we found before and during COVID-19 confinement. The literature supports these results, as eating styles tend to be considered as a trait rather than state factors [43] and often show temporal stability in the young population despite adverse circumstances [44], as well as in the clinical population [43]. Therefore, our result reinforces the idea that eating attitudes have a stable character, even after experiencing stressful events, such as COVID-19 confinement.

The second objective was aimed at analyzing whether the maladaptive eating styles assessed before COVID-19 confinement influenced binge eating, fat intake, and BMI during COVID-19 confinement. The scores revealed that emotional eating positively predicted BMI and binge eating but did not predict fat intake. This result can be explained by the classical psychosomatic theory, in which people who exhibit emotional eating cannot differentiate hunger from physiological signals accompanying negative emotions [45]. Therefore, individuals respond to negative emotions by eating more [46]. The emotional eating also predicted the increase in BMI, confirming longitudinally the strong relationship found in the literature [47, 48].

In the present study, the previous emotional eating influenced only binge eating and not fat intake. It can be possible that unhealthy food consumption did not occur in response to negative emotions in this case [49]. Another hypothesis could be that emotional eating may be associated with impulsivity behavior and loss of control over food intake in the face of negative emotions, using this behavior to regulate the emotions. In this case, it might not influence what someone eats (e.g., unhealthy food) but how much it is eaten (e.g., binge eating). Future studies are needed to further analyze which personal factors predispose to emotional eating.

Our results also indicated that external eating positively and marginally predicted fat intake during COVID-19 confinement, although it did not predict binge eating and BMI. The first result supports the idea of other studies, which indicate that external eating is often associated with higher food intake, principally high-fat foods [23]. However, external eating did not predict binge eating during COVID-19 confinement. Although external eating style may serve as a predisposing factor for binge eating, this seems to occur in combination with high BMI and depressive problems [15]. Regarding the non-significant effect of external eating on BMI, this was consistent with the results of another study [23]. Hence, this finding imply that an increase in BMI in our sample may be determined to a greater extent by emotional eating than by the response to environmental cues [50]. However, considering that most of our participants had a normal weight and the sample was nonclinical, it seems plausible that this relation was no significant. Moreover, it should be noted that the BMI variability of our sample is limited, with underrepresented BMI ranges, which could be biasing this relationship. Furthermore, the relationship between external eating and BMI could have been mediated by the craving for fatty food and fast food [51]. Future studies should analyze whether the craving influences this relationship during long periods of confinement since only the direct relationship between external eating and BMI was studied in our study.

Finally, our results showed that restrained eating positively predicted binge eating, and negatively predicted fat intake and BMI during COVID-19 confinement, although the latter result was marginal. These results are consistent with the fact that restrained eaters want to lose or maintain weight, leading them to eat less fat to accomplish this goal [23]. However, calorie deprivation may precipitate binge eating, because of the loss of cognitive control and the inability to maintain the diet [52, 53].

Hence, this study indicates that eating styles may be key determinants in understanding disordered eating behaviors and increased BMI during long periods of stress (e.g., confinement). Consequently, some practical clinical implications should be highlighted. Thus, emotional, restrictive, and external eating styles are relatively stable eating styles that can be identified in order to develop vulnerability profiles to make programs for preventing weight gain, binge eating, and fat intake during chronic stress. Moreover, this is especially relevant in women, since altered eating styles (e.g., emotional eating) are especially high in women, making them more vulnerable to have less healthy eating behaviors [54]. In this regard, health policies should study and early detect, on a large scale, the harmful effects of dysfunctional eating styles on disordered eating behaviors (e.g., binge eating, fat intake) and weight gain.

Our results suggest that interventions focused on reducing dysfunctional eating behaviors in youth at times of high stress could focus on providing cues to control emotional hunger in youth (e.g., mindful eating techniques) and on controlling or reducing any external or restraint behaviors that may lead to increased binge eating, fat intake, or BMI. However, these results should be interpreted with caution given the small sample size of the present study. Future studies should try to analyze, in a larger sample, the causal relationship between different eating behaviors to prevent and treat obesity and health problems in young people.

### Strengths and limitations

This study has some limitations. One weakness is that body weight was self-reported, which could lead to underestimating or overestimating the data. Besides, measures of eating behaviors were also self-reported, which may lead to biased results compared to more structured clinical assessments or interviews [55]. Another limitation is related to the sample size of the study, as it was composed of college students, and its size was relatively small. To increase the representativeness of the study, future studies should include a larger sample and include other populations, such as older adults or clinical populations (e.g., people with obesity or people with eating disorders). Despite these limitations, our study also presents several strengths. It is a longitudinal study that examines dietary variables and BMI before and during COVID-19 confinement; so, the present study is not biased by the common limitations of the retrospective studies. In addition, it is the first study to assess the predictive role of maladaptive eating styles on binge eating, fat intake, and BMI in a highly stressful context.

## Conclusions

COVID-19 confinement may have a positive impact on youths' eating behaviors. In addition, it has been shown that emotional, external, and restrained eating can directly influence binge eating, fat intake, and BMI during the COVID-19 confinement. Most people experienced COVID-19 confinement as a stressful situation, which implied a sort of characteristics that are specifics for this situation (e.g., the movement restriction), but with some commonalities with other stressful situations (e.g., the uncertainty about the future, the emotional dysregulation). In this sense, results obtained in the study could be extended to other situations and could lead to future studies, where preventing or treating maladaptive eating styles may help preventing eating and weight related problems.

## Public significance statement

Previous cross-sectional studies assessed the changes in dysfunctional eating behaviors and BMI during COVID-19 confinement, but no studies assessed longitudinally these variables before and during COVID-19 confinement. This longitudinal study shows the changes in binge eating, fat intake, BMI, and maladaptive eating styles (before and during COVID-19 confinement in Spain), and the predictive role of maladaptive eating styles (before COVID-19 confinement) on binge eating, fat intake, BMI (during COVID-19 confinement) in college students.

## Data Availability

The datasets generated during and/or analyzed during the current study are available in the OSF repository, https://osf.io/qfc7d/?view_only=85911ed7b2e24a5ab16447a887b90520

## Abbreviations

BES: Binge eating scale
BMI: Body mass index
CFI: Comparative fit index
DEBQ: Dutch Eating Behavior Questionnaire
M: Mean
MLR: Maximum likelihood robust
SD: Standard deviation
T1: Time 1, before confinement
T2: Time 2, during confinement
SEM: Structural equation model
SFQ: Short fat questionnaire
SRMR: Standardized root mean square residual
WLSMV: Weighted least squares mean and variance corrected

## Footnotes

1 BMI was calculated following WHO guidelines [26].
